# Prevalence of Shiga-Toxigenic *Escherichia coli* in Bovine Manure in the Mid-Atlantic Region of the United States

**DOI:** 10.3390/microorganisms13020419

**Published:** 2025-02-14

**Authors:** Pushpinder K. Litt, Alexis N. Omar, Samantha Gartley, Alyssa Kelly, Thais Ramos, Esmond Nyarko, Tenille Ribeiro de Souza, Michele Jay-Russell, Yuhuan Chen, Peiman Aminabadi, David T. Ingram, Kalmia E. Kniel

**Affiliations:** 1 Department of Animal and Food Sciences, University of Delaware, Newark, DE 19716, USA; 2 Western Center for Food Safety, University of California, Davis, CA 95616, USA; 3 Center for Food Safety and Applied Nutrition, U.S. Food and Drug Administration, College Park, MD 20740, USA

**Keywords:** STEC, prevalence, enumeration, *stx*, Clermont PCR, bovine manure, dairy cattle, mid-Atlantic

## Abstract

Shiga toxin-producing *Escherichia coli* (STEC) is a foodborne pathogen and known to reside naturally in cattle. The application of untreated biological soil amendments of animal origin on fresh produce fields results in unique food safety challenges. It is critical to identify farm manure management practices to mitigate pre-harvest pathogen contamination. The objective of this study was to quantify the prevalence and level of STEC in cattle manure in the Mid-Atlantic region of the United States. A total of 161 bovine manure samples were collected from 13 cattle farms between 2016 and 2018. The samples were enriched with non-selective and selective media and quantified following a Most-Probable Number (MPN) assay. Among the recovered STEC isolates, PCR was performed to determine the presence of *stx*, *eae*, and *rfbE*. Clermont PCR was performed to identify phylogenetic groups of isolates. Of the 13 farms, 11 had STEC populations between <1.0 and >5.6 log MPN/g. Farm, humidity, and sampling year significantly (*p* < 0.05) influenced STEC populations in bovine manure. Of the 108 isolates, 50% were *stx+* and 14% *eae+*. Phylogenetic group analysis revealed that 46% of the isolates belonged to group A, 19% to B1, 7% to B2, and 28% to D. Group D had the highest prevalence of *stx+* and *eae+* and group B1 had the lowest prevalence. Results suggest STEC geographical distribution in the Mid-Atlantic region is farm-specific, and climatic conditions can be critical for its survival and dissemination.

## 1. Introduction

The incidence of foodborne illness remains a serious public health concern. Mitigation of risks of foodborne illness from contaminated fresh fruits and vegetables, which collectively are known as fresh produce, is a component of a One-Health Approach to enhancing food safety and public health [1]. According to the Centers for Disease Control and Prevention (CDC), approximately 48 million people become sick each year due to foodborne illnesses in the United States (U.S.) [2]. In 20% of these cases (9.4 million illnesses), the causative agents (pathogens) can be identified, imposing an annual economic burden of over USD 15.5 billion [3]. Most of the economic burden (84%) is associated with pathogen-related deaths. Shiga toxin-producing *Escherichia coli* is associated with 175,905 cases of foodborne illnesses each year in the U.S. and is estimated to impose an economic burden of approximately USD 50 million in medical costs [3]. In the U.S., from 2010 to 2015, 942 outbreaks were attributed to STEC, of which 32% were foodborne and 48 of those involved fresh produce [4]. Recently, multiple multistate STEC associated outbreaks have been reported. In 2018, *E. coli* O157:H7-linked outbreak caused 210 illnesses in 34 states, 94 hospitalizations, and five deaths, and was traced back to romaine lettuce grown in Yuma, Arizona [5]. In 2019, 167 individuals were infected with *E. coli* O157:H7, and cases were reported from 27 states with 85 hospitalizations and no deaths. Traceback investigation indicated that Romaine lettuce grown in Salinas Valley, California was likely the source [6].

Shiga toxin-producing *E. coli* are defined by the presence of shiga toxin-encoding *stx* genes from a temperate phage. The presence of an intimin protein-encoding *eae* gene on the locus of the enterocyte effacement (LEE) pathogenicity island is required for intestinal colonization and intimate attachment [7]. *E. coli* O157 is the prototype serogroup of the STEC pathotype, but six other serogroups (O26, O45, O103, O111, O121, and O145) are also emerging as human pathogens; in combination, these serogroups are referred to as the “top 7” and are considered to be adulterants in beef by the U.S. Department of Agriculture (USDA) Food Safety and Inspection Service [8]. Shiga toxin can be divided into two groups: Stx1 and Stx2 with additional subtypes (e.g., Stx1a, c, d, and Stx2a to Stx2g) [9]. Bacterial isolates containing the LEE island and at least one *stx* subtype represent a subset of STEC strains that are classified as enterohemorrhagic *E. coli* (EHEC). Although some STEC strains of various serotypes are capable of causing hemorrhagic colitis and hemolytic uremic syndrome (HUS), EHEC typically causes more severe clinical symptoms in humans [7,10].

Several studies have linked sporadic or repeated contamination events in and around fruit and vegetable fields to wildlife fecal deposits [11,12,13], with a variety of bacterial foodborne pathogens, including STEC, regularly isolated from fecal samples collected from wildlife and domesticated animals [13]. Ruminants, especially cattle, are recognized as a major reservoir for STEC. It is estimated that about 30% of the cattle shed these pathogens in their feces [14], which could lead to pathogen dispersal and contamination of untreated drinking water, surface water including irrigation reservoirs, and potential transfer to human food crops. In 2017, an outbreak associated with STEC O157:H7 illnesses was reported in Utah and Arizona, and the outbreak strain was isolated from bull and horse manure collected from a yard near a community household with two ill children [15]. In 2006, an environmental investigation, following a multistate outbreak of *E. coli* O157:H7 associated with bagged baby spinach, identified the outbreak strain in feces from an adjacent “grass-fed beef” cow-calf herd and free-roaming feral swine in the central California coast [16,17]. Manure from cattle feedlots could generate airborne particulates, which have the potential to contaminate lettuce plants with STEC O157:H7 from a distance of 180 m (590 ft) [15,16]. In 2018, an environmental investigation followed a multistate outbreak of *E. coli* O157:H7 on Romaine lettuce [18]. Water samples were collected from an irrigation canal in the Yuma produce growing region in Arizona, and they were genetically similar to the environmental isolates of *E. coli* O157:H7 found by an adjacent cattle ranch [19]. Windborne transmission of contaminated dust from the adjacent cattle farm onto Romaine lettuce crops or canal water is speculated to be the source of contamination [19]. Moreover, the contaminated dust has been reported to support longer survival durations of bacterial pathogens on leaves of spinach plants, compared with waterborne contamination [13].

Mitigating contamination risks at the pre-harvest level can be complex and challenging. Modifying manure management practices, such as covering the manure piles, installing fences, addition of buffer zones, and controlling nuisance pests such as rodents, birds, and filth flies, has the potential to reduce on-farm contamination events. However, some of these practices and geospatial factors (soil characteristics) could significantly modulate the risk of contamination [20]. STEC can survive in the soil for long durations when introduced by contaminated manure [21]. Recent documents provide information regarding mitigation of the spread of bacterial pathogens through the use of raw soil amendments. The FDA Food Safety Modernization Act (FSMA), specifically the Produce Safety Rule (PSR) Subpart F, focuses on the use of biological soil amendments of animal origin (BSAAO), which is defined as “a biological soil amendment which consists, in whole or in part, of materials of animal origin, such as manure or non-fecal animal byproducts including animal mortalities, or table waste, alone or in combination” [22]. FDA also released the 2020 Leafy Greens Action Plan to address the recurring nature of outbreaks associated with leafy greens [23]. While several studies [24,25,26,27,28] have assessed STEC prevalence in soil and manure, a knowledge gap exists in quantifying STEC in manure samples from multiple farms in several geographical regions and is associated with different manure management practices, which could impact pathogen contamination in the pre-harvest environment. These types of data are critical for risk assessment relevant to FSMA Produce Safety Rule provisions and are essential to allow for the identification of environmental factors and practices that can significantly affect STEC survival to facilitate the implementation of science-based preventive controls. Thus, the purposes of this study were to (i) evaluate the prevalence and level of STEC in bovine manure from different dairy farms in the Mid-Atlantic region and (ii) identify manure management practices and environmental factors associated with STEC prevalence.

## 2. Materials and Methods

### 2.1. Sample Collection

Manure samples (*n* = 161) were collected from 13 dairy farms, each small- to mid-sized with approximately 50–150 cattle, between June 2016 and June 2018 in and around Delaware. The farms were visited at random. The sampled manure was stored at the farm as piles (stacked manure) or a lagoon. Seven bovine manure samples were collected from each farm during each sampling, using a stratified sampling method similar to that used in parallel survey of STEC in untreated manure in the southeast [29] and on the west coast of the U.S. [30]. From a pile, two samples were collected from surface and five subsurface, at every subsequent 12 inches, and from a lagoon, all samples were collected from the surface. The collected samples were transported to the laboratory on ice in a cooler and processed within 24 h. From each sampling location, the following information was collected: facility type, cattle type, and manure type, herd size, manure storage type (pile or lagoon), number of manure storage location at a facility, whether the manure pile was covered, size and age of the pile, sample depth, sample temperature, humidity, rainfall event, and moisture content. Air temperature and relative humidity of 5 days before sampling was obtained from the University of Delaware Weather Station, available at http://www.deos.udel.edu/data/monthly_retrieval.php, accessed on 9 December 2024.

### 2.2. Sample Enrichment and STEC Isolation

The samples were processed as described by Baker et al. [29]. Each manure sample (30 g) was enriched in 270 mL of tryptic soy broth (TSB; Bacto™, BD, Sparks, MD, USA) using a 24 oz Whirl-Pak™ bag (Nasco, Fort Atkison, WI, USA). The enriched samples were hand-massaged for 1 min and incubated at 25 °C for 2 h, followed by 12 h at 42 °C on a shaking incubator at 50 rpm. After incubation, 1 mL suspension was transferred to a tube containing 9 mL of mEHEC broth (Difco™, BD, Sparks, MD, USA) and incubated for 48 h at 42 °C. On the following day, 10 µL from the enrichment was struck onto STEC CHROMagar (CHROMagar Microbiology, Paris, France) plate and incubated at 37 °C for 24 h. The plates were observed for presumptive positive STEC colonies and purified by re-streaking. The frozen stocks of STEC isolates were prepared and stored at −80 °C until further use. The presumptive positive samples were confirmed by performing PCR for the presence and absence of *stx-1* and *stx-2* genes and evaluated to quantify STEC populations using MPN assay [31].

### 2.3. Determination of MPN/g for Potentially Positive Samples

To quantify presumptive positive samples, prior to the initial incubation of the above prepared manure-TSB slurry (30 g manure + 270 mL TSB), 5.5 mL of the slurry was transferred simultaneously to the first four wells (as four replicates) of the first column of the 48 well reservoirs. The sample was serially diluted to 10^−6^ of the manure sample in TSB by transferring 0.5 mL of the sample from the previous column to respective columns of 4.5 mL TSB using a multi-channel pipette. The reservoir was sealed using an adhesive film (VWR, Philadelphia, PA, USA) and incubated at 25 °C for 2 h with 50 RPM shaking followed by 42 °C for 12 h with 50 RPM shaking. After incubation, 0.5 mL of the enrichment from the TSB reservoir was transferred to respective wells in a reservoir containing 4.5 mL of mEHEC broth. The reservoir was sealed and incubated at 42 °C for 12 h and held in refrigeration until STEC presence was determined from sample enrichment and STEC isolation. The presumptive positive samples were channel-streaked (5 µL) on STEC CHROMagar plates to quantify STEC populations in the samples. The plates were incubated at 37 °C for 24 h, and positive streaks/colonies were recorded. The log CFU MPN/g calculations were performed using an MPN calculator, available at http://standards.iso.org/iso/7218/, accessed on 9 December 2024 [3].

### 2.4. Prevalence of stx1/stx2, eae, and rfbE in STEC Recovered from Bovine Manure Samples

DNA lysate was prepared by following the heat and cold shock method. Briefly, a loopful of presumptive positive colonies was suspended in 50 µL of DNase-free water in a sterile 1.5 mL centrifuge tube and vortexed for 10 s. The tube was placed in a water bath at 100 °C for 20 min, followed by 20 min at −20 °C freezer. The lysate was used to screen presumptive STEC colonies for the presence of *eae*, *stx-1*, and *stx-2* genes through PCR.

The reaction mixture of 25 µL was prepared by adding 2 μL of DNA, 12.5 μL of PCR master mix (Thermo Scientific™ DreamTaq™, Lafayette, CO, USA), 1 μM of *eaeF* (AAAGCGGGAGTCAATGTAACG), *eaeR* (GGCGATTACGCGAAAGATAC) *Stx-1F* (ATAAATCGCCATTCGTTGACTAC), *Stx-1*R (AGAACGCCCACTGAGATCATC) and *Stx-2F* (GGCACTGTCTGAAACTGCTCC), and *Stx-2R* (TCGCCAGTTATCTGACATTCTG) primers, as well as 8.7 µL of sterile DNase-free water (Thermo Scientific™, Lafayette, CO, USA). The multiplex PCR reaction was performed under the following conditions: initial denaturation at 95 °C for 20 s 40 cycles of denaturation at 95 °C for 30 s, annealing at 60 °C for 30 s, extension at 72 °C for 60 s, and final extension at 72 °C for 5 min, followed by a 4 °C hold. For *rfbE*, the reaction mixture of 25 µL was prepared by adding 2 μL of DNA, 12.5 μL of Green Taq, 1 μM of *rfbE* F (CTGTCCACACGATGCCAATG), and *rfbE* R (CGATAGGCTGGGGAAACTAGG) primer, and 9.5 µL of sterile DNase-free water. This PCR reaction was performed under the following conditions: initial denaturation at 94 °C for 5 min, 40 cycles of denaturation at 94 °C for 30 s, annealing at 65 °C for 20 s, extension at 68 °C for 75 s, and final extension at 68 °C for 7 min, followed by a 4 °C hold.

### 2.5. Phylogenetic Analysis of STEC Isolates Recovered from Bovine Manure Samples

STEC isolates phylogenetic analysis was performed by testing for the presence of three genes, including *chuA*, *yjaA*, TspE4C2.2 [32]. The reaction mixture of 25 µL was prepared by adding 2 μL of DNA, 12.5 μL of Green Taq, 1 μM of *chuA*-F (GACGAACCAACGGTCAGGAT), *chuA*-R (59-TGCCGCCAGTACCAAAGACA-39), *yjaA*-F (TGAAGTGTCAGGAGACGCTG), *yjaA*-R (ATGGAGAATGCGTTCCTCAAC) and TspE4C2.2-F (GAGTAATGTCGGGGCATTCA), and TspE4C2.2-R (CGCGCCAACAAAGTATTACG) primers, as well as 4 µL of sterile DNase-free water. A multiplex PCR was performed with a thermal cycler under the following conditions: denaturation for 5 min at 94 °C; 30 cycles of 30 s at 94 °C, 30 s at 55 °C, and 30 s at 72 °C; and a final extension step of 7 min at 72 °C.

### 2.6. Statistical Analysis

The prevalence was determined as the number of positive samples for each gene divided by the total number of samples evaluated. Fisher’s exact tests was performed to compare the effect of herd size, manure storage type (pile or lagoon), number of manure storage location at a facility, whether the manure pile was covered, size and age of the pile, sample depth, sample temperature, humidity, rainfall, and moisture content on STEC survival or recovery, the presence of *stx*, *eae*, and phylogenetic group composition. A significance level (*p* < 0.05) was used for all analyses. All statistical analyses were performed using JMP Pro 14 (SAS Institute Inc., Cary, NC, USA).

## 3. Results

In total, 161 samples were collected from 13 farms between 2016 and 2018. Among the 13 farms tested, 11 were presumptive positive for non-O157 STEC. Overall, farm, humidity, and sampling year were significant (*p* < 0.05) factors in differences in the recovery of STEC, whereas manure pile number, pile height, sample depth, pile temperature, pile age, pile size, pile width, pile length, heard size, air temperature, rainfall, sample weight, and moisture content were not significant (*p* < 0.05) factors in STEC survival.

### 3.1. Determination of MPN/g for Potential STEC Positive Bovine Manure Samples

Among the 161 bovine manure samples collected from 13 farms, 26 samples (11 farms) were presumptive STEC positive, based on the results using an MPN assay. The majority of STEC presumptive positive samples had populations between 1.0 and 6.6 log MPN/g (Table 1). Most presumptive positive samples were collected during summer months of the year. These results align with previous studies suggesting higher STEC recovery during summer months compared to winter or fall season [33].

STEC loads varied from farm to farm, and the farm itself was a significant (*p* < 0.05) factor in differences in STEC populations, suggesting that larger or smaller heard size did not affect the number of positive samples identified from a particular farm. However, humidity and sampling year showed a significant (*p* < 0.05) effect on STEC survival. As the humidity increased, STEC populations increased significantly (*p* < 0.05). Samples collected on a day documented high relative humidity of 100% and had significantly higher STEC populations (6.6 logs MPN/g) compared to a day of 76% relative humidity with populations of 1.3 log MPN/g. Across farms, there was no significant (*p* < 0.05) correlation between presumptive STEC positives and pile type (lagoon or stacked manure), sample depth (0, 12, 24, 36, 48, 60 inches), pile temperature, rainfall, pile size, air temperature, and moisture content.

### 3.2. Prevalence of stx1/stx2, eae, and rfbE in STEC Recovered from Bovine Manure Samples

STECs were assessed for the presence of *stx1*, *stx2*, *eae*, and *rfbE* genes (Table 2). The results show a prevalence of 23% (25/108) for *stx1* and/or 28% (30/108) for *stx2*, 14% (15/108) for *eae* and 4% for *rfbE* in isolates identified from bovine manure. Of these isolates, three were detected to have *stx1+/stx2+/eae+*; 16 *stx1+/stx2+*; 6 *stx2+*/*eae+*; 6 *eae+*; 6 *stx1*+; and 5 *stx2*+. For *stx*, 16% of the positive isolates had *stx1+/stx2+*, while the rest had either *stx1+* or *stx2+* isolates. For *eae*, 6% of positive isolates had *eae+* alone, while 6% were also positive for *stx2*+, and 3% also for *stx1+/stx2+*. For *rfbE+*, 50% of the positive isolates were also carrying *stx1+/stx2+*, while 25% also had either *stx1+/stx2+/eae+* or *eae+*. The highest percentages of *stx* (*stx1 and stx2;* 22%), *eae* (9.3%), and *rfbE* (2.8%) were observed in sample group 311; this could be due to higher numbers of positive samples being identified from this group (Table 2). Manure storage type, pile temperature, humidity, moisture content, and sampling season did not play a significant role in *stx* or *eae* gene prevalence. However, *eae* prevalence varied significantly among farms and sampling years.

### 3.3. Phylogenetic Analysis of the STEC Isolates Recovered from Bovine Manure Samples

A total of 108 presumptive STEC positive isolates were collected from 28 positive samples. Of these 108 isolates, the predominant phylogenetic group was A (50/108; 46%), followed by B1 (20/108; 19%), B2 (8/108; 7%), and D (30/108; 28%) (Table 2). Phylogenetic-typing analysis revealed that isolates (*n* = 3) carrying all three genes (*stx1*, *stx2*, *eae*) belonged to phylogroup D. Group D also had isolates with *stx1+/stx2+* (13/30; 43%), *stx2+/eae+* (5/30; 17%), along with isolates carrying only *stx2+* (5/30; 17%), or *eae+* (2/30; 7%) or *rfbE+* (3/30; 10%). Group D had one isolate, which amplified all four genes (*stx1*, *stx2*, *eae*, and *rfbE)*, suggesting virulent or pathogenic strains might belong to this phylogroup. Group B2 isolates had *stx1+/stx2+* (4/8; 50%) or *stx1* (1/8; 13%) or *stx2+/eae+* (1/8; 13%). Group B1 isolates only carried either *eae+* (3/20; 15%), or *stx1* (2/20; 10%) or *rfbE+* (1/20; 5%). Group A isolates had either *stx1+*(3/50; 6%) or *eae*+ (1/50; 2%). All 50 isolates of group A and 20 isolates from group B1 lacked *stx2* genes. The results indicate that group A appears to be a non-virulent phylogroup, with the absence of *stx2* and only one *eae+* isolate. While minor variations in significance were observed in *stx2* and phylogenetic group composition, no significant (*p* < 0.05) difference was determined within the phylogenetic grouping of isolates with *stx1* and *eae*.

The results show that significantly (*p* < 0.05) higher group D isolates were identified in 2016 compared to group A and B2. This could be due to the higher sampling frequency in 2016 than in 2017 or 2018. During fall sampling, significantly (*p* < 0.05) higher group B isolates were recovered compared to summer or winter months. Further analysis showed that a significantly (*p* < 0.05) higher number of isolates recovered from subsurface samples (depth = 36 inches) belonged to group B2 compared to group A or B1. At levels of high relative humidity (88–92%), a significantly higher (*p* < 0.05) number of recovered isolates belonged to groups B2, A, and D, compared to group B1. Farm conditions like storage type (lagoon or stacked manure) and pile temperature did not significantly affect the phylogenetic group of isolates.

## 4. Discussion

This study reported the prevalence and level of STEC in bovine manure in the Mid-Atlantic region of the United States and the association with environmental or farm management factors. The results show that, of the 13 cattle farms studied, 11 farms had STEC-positive manure samples containing populations between <1.0 and 6.6 log MPN/g. From these 11 farms, 108 isolates were recovered, and 50% of the isolates were *stx+* and 14% *eae+*. Manure samples from only two farms had *rfbE*+ isolates. Among all bovine manure samples collected at farms with at least one STEC-positive sample, prevalence rates of 3% (3/108) for *stx1+/stx2+/eae+*; 15% (16/108) for *stx1+/stx2+*; 6% (6/108) for *stx2+*/*eae+*; 6% (6/108) for *stx1*+; 5% (5/108) for *stx2*+; 6% (6/108) for *eae+*; and 4% (4/108) for *rfbE*+ were observed. Phylogenetic group analysis revealed that 46% (50/108) of the isolates recovered from STEC-positive manure samples belonged to group A, 19% (20/108) to B1, 7% (8/108) to B2, and 28% (30/108) to D. In the analysis, three factors (farm, humidity, and sampling year) were identified that significantly (*p* < 0.05) influenced STEC recovery in bovine manure samples.

Prior studies have reported that several other factors could contribute to STEC prevalence and persistence in an environment or host. For example, geographical location (different farm locations) and environmental or climatic factors (temperature, rainfall, and relative humidity) have been shown to impact STEC survival and persistence [29,34,35]. Studies have revealed that STEC prevalence varies by geographic region [34,36,37]. Benjamin et al. [28] conducted a 2-year study of STEC O157 prevalence in rangeland herds in the Salinas Valley and found relatively low prevalence, but periodic spikes and variation by weather conditions and herd size. Another study conducted in the Salinas Valley area of California reported the seasonal prevalence of STEC in non-irrigation watersheds and found that the overall prevalence of STEC strains was lower during summer and fall periods [38]. In another study, authors [36] discovered the geographical distribution of STEC plays a significant role in its prevalence compared to specific farm practices in Ohio (US) and Norway. Similarly, in the present study, we found seasonal variation; most of the STEC positive samples were recovered in the summer months. We also found that farm management factors did not significantly affect STEC prevalence, including manure storage type (pile or lagoon), number of manure storage locations at a facility, manure pile covering, size, and age of the pile (fresh or ranging from 1 to 8 days for 91% of the samples where information was available). Furthermore, we quantified the level of STEC in each of the positive samples and found that relative humidity significantly influenced the STEC concentration in the samples.

Based on the analysis of the STEC prevalence and concentration data, differences in prevalence were observed across farms with positive manure samples, with significant variation being detected between the sampling years and humidity levels. It has been previously reported that higher frequencies of STEC are recovered in the summer months [33,39,40,41,42,43]. Other studies have shown that during summertime, a higher prevalence of STEC specific bacteriophage populations might account for the low prevalence of STEC bacterial isolates [44,45]. These are plausible reasons supporting our finding that most of the positive samples recovered in the current study were from summer sampling.

Relative humidity was another significant factor in STEC survival in the current study. Increased relative humidity in the environment was associated with increased populations of STEC and their prevalence, suggesting a seasonal effect on STEC survival. Previous studies have found an association between higher humidity levels with increased risk of shedding STEC in manure [35,46]. Williams et al. [35] showed a significant association of rainfall and higher humidity levels with increased shedding of *E. coli* O157: H7 in the manure of 52 super shedder dairy cattle. High relative humidity are associated with higher moisture and temperatures, which could provide optimal growth conditions for STEC in the environment. Moreover, it is speculated that changing relative humidity or climate conditions could set a stress response in cattle, affecting the shedding of the pathogen in manure [25].

Studies have evaluated variations in the *stx* gene distribution [29,47,48]. Among the positive samples in the current study, the *stx* (50%) and *eae* (14%) genes predominated in bovine manure from all but one sample. The majority (28%) of the isolates identified from these samples were *stx2*+ followed by *stx1* (23%) and *eae* (14%). In this study, 15% of the *stx* positive isolates were detected to have *stx1+/stx2+* genes, and 10% isolates harbored *stx1 or stx2* genes. These results are in agreement with previous studies [47,48,49]. Venegas-Vargas et al. [48] documented that 42% of the isolates recovered from animals were positive for *stx2* genes, and 29% had either *stx1* or *stx2*. Similarly, a separate study reported a prevalence of 40% *stx*2+ gene among isolates recovered from dairy farms in the Eastern Cape Province of South Africa, and 10% had *stx*1 and *stx*2 [50]. Moreover, the specific reasons for the higher prevalence of *stx2+* isolates were not identified. Other studies have suggested that changes in diet and handling of animals in different seasons can have an impact on the gene selection of strains [33,51]. In addition, *stx* is located in the mobile genetic elements of the bacterial genome and could impact its mobility patterns [51]. These results are of importance, given that *stx*2-linked infections have been associated with the more severe disease outcomes in humans [52,53].

Likewise, 14% of the isolates were positive for *eae*, and their prevalence varied significantly across farms and sampling year. The *eae* gene is located in the LEE (locus of enterocyte effacement) pathogenicity island and has also been linked to enhanced virulence and attaching and effacing (A/E) lesions [10]. In the current study, of the isolates identified from different farms carrying *eae*, 2.8% also carried *stx1+/stx2+*, 6% also carried *stx2+*, and only 6% carried *eae* only genes. These findings are consistent with previous studies, which reported a lower frequency of *eae+* isolates in bovine manure [26,27,54]. Oporto et al. [26] documented that 8.1% (30/247) *eae+* isolates (non-O157 STEC) recovered from the ruminant manure samples collected from northern Spain. In a different study, Monaghan et al. [54] reported that 17% (18/108) of the isolates recovered from bovine manure samples in Ireland contained *eae* genes. The presence of isolates carrying only the *eae* gene suggests the occurrence of atypical enteropathogenic *E. coli* (aEPEC) strains in the bovine manure, which could cause severe human diseases [10], resulting in sporadic cases or small outbreaks [24]. Although it has been suggested that in the absence of *eae*, STEC isolates are unlikely to be associated with large outbreaks and HUS [55,56], an *eae* negative O104: H4 strain was still linked with an outbreak in Germany in 2011 [57].

Moreover, in the current study, only two sampling groups had four isolates carrying *rfbE*+ isolates. The *rfbE* gene encodes for O antigen (O157) of isolates. It has been suggested that testing for *rfbE*, along with *stx* and *eae* genes, can be effective in the detection of O157 STEC in bovine manure samples [58,59]. These results indicate that bovine manure samples collected from the Mid-Atlantic region of the U.S. are primarily contaminated with non-O157 STEC. These findings are consistent with previous studies, illustrating the higher prevalence of non-STEC in dairy cattle compared to O157 STEC [33,36]. These studies reported serogroup O157 prevalence between 0.2% and 0.8% in dairy cattle [33,36]. Similarly, in the current study, 3.7% of isolates were able to amplify the O157 antigen encoding gene (*rfbE*). Based on epidemiological data, the severity of human infections caused by STEC groups. The isolates recovered in the present study will be further characterized in the future to assess prevalence frequencies by serotype through whole-genome sequencing.

Clermont PCR was performed to assess a relationship between phylogenetic groups and gene status as *stx*+/− and *eae*+/−. In the current study, the highest number of isolates identified belonged to group A, D, and B1. Similar results have been reported previously by Glaize et al. [60], where the authors found that the highest number of *E. coli* isolates that recovered from raw manure were categorized in phylogroup A, B1, and D. In the current study, 35% of the total isolates characterized belonged to either phylogroup B2 or D, which are more likely to be associated with extraintestinal infections in humans [61,62]. On the other hand, isolates from phylogroups A and B1 are considered generic or commensal *E. coli* and can be found in the environment [63]. This could also explain the highest prevalence of virulent genes (*stx* and *eae*) in group D isolates and the lowest prevalence in group B1 in the present study.

In the current study, sampling groups 311 and 412 had the highest number of isolates, representing phylogroup D (14 and 9, respectively), while 211 had the highest number of isolates from group B2. This indicates that more cattle might be shedding STEC, or the geographical location of the sampling site affects *E. coli* distribution. Studies have suggested an association between specific phylogroup prevalence and association with different ecological niches [31,60,63,64,65]. A study by Howard et al. [66] indicated that wildlife intrusion at any stage of the manure management system on-farm could be a factor in the increased prevalence of phylogroup B2. Moreover, significantly (*p* < 0.05) higher group B2 isolates were recovered from the subsurface samples (depth = 36 inches), compared to group A or B1. This could be due to the composition of the natural microbial community of the manure pile sampled. It has been suggested that the microbial diversity of the stored manure could influence the prevalence or survival of pathogens [67]. However, in the present study, manure community analysis was not performed. Including community analysis in future, manure management practices could provide additional information on the survival and transmission of pathogenic microbes on farms.

## 5. Conclusions

This study provides quantitative data on the STEC prevalence and level in bovine manure in the Mid-Atlantic region of the United States. This study indicates that environmental temperature, rainfall, sample depth, whether the pile was covered, and manure storage type did not affect pathogen detection in the bovine manure samples. Relative humidity could impact STEC prevalence in bovine manure. This dataset pertains to a specific geographical region of the country. In different areas, geographic location, farm practices, and environmental factors could influence STEC prevalence. Virulent gene distribution and phylogenetic group analysis provide valuable information about the genetic diversity of STEC found in dairy farms of the mid-Atlantic region of the country. This information could provide an opportunity to improve control strategies, such as manure treatment, to minimize on-farm contamination in produce production and pathogen dissemination in the environment. Moreover, these data may inform risk assessments to evaluate the risk of human illness associated with the use of untreated biological soil amendments of animal origin (e.g., raw bovine manure) for the growth of fresh fruits and vegetables, as well as studies to estimate the risk of cross-contamination associated with adjacent land usage.

## Figures and Tables

**Table 1:** STEC populations (MPN/g) in bovine manure samples based on sample number, sample location, depth and humidity. ^†^ Limit of detection is −0.05 log MPN/g.

Sample No.	Sample Depth (cm)	Relative Humidity (%)	Log MPN/g ^†^
111.6	Surface (Lagoon)	79	5.00
111.7	Surface (Lagoon)	79	4.28
112.1	Surface (Lagoon)	79	2.52
112.2	Surface (Lagoon)	79	2.34
113.1	30.48	79	2.98
211.1	30.48	91	1.26
211.3	91.44	91	2.90
211.4	121.92	91	2.04
213.1	30.48	91	1.14
213.3	91.44	91	4.11
214.2	60.96	91	2.90
311.1	30.48	100	>5.62
311.2	60.96	100	>5.62
311.3	91.44	100	>5.62
311.4	121.92	100	>5.62
313.4	91.44	92	>5.62
313.3	Surface	50	−0.05
314.2	60.96	92	>5.60
314.4	121.92	92	3.38
314.5	Surface	50	3.11
314.6	Surface	92	>5.60
411.6	Surface	76	0.26
412.7	106.68	42	−0.37
412.2	60.96	76	2.08
412.3	91.44	76	3.66
412.7	Surface	76	−0.25

**Table 2:** STEC isolates for the presence of *stx+*, *eae+*, and *rfbE*+, and phylogenetic classification recovered from bovine manure based on samples.

Sample Group	Phylogenetic Classification				*stx1+ stx2+ eae+*	*stx1+/stx2+*	*stx2+/eae+*	*stx1+* Only	*stx2+* Only	*eae+* Only	*rfbE+*
	A	B1	B2	D							
111	4	0	0	4	0	1	2	0	0	0	0
112	9	4	0	1	0	0	1	1	0	0	0
211	12	1	7	0	0	3	1	2	0	0	0
213	4	0	0	0	0	0	0	0	0	0	0
311	8	5	1	14	3	7	2	1	1	5	3
314	8	2	0	1	0	0	0	1	1	1	1
411	5	8	0	1	0	1	0	1	0	0	0
412	0	0	0	9	0	4	0	0	3	0	0

## Data Availability

The data presented in this study are available on request from the corresponding author due to associated legal issues.
